# Modernizing public health surveillance for global health security leveraging AI

**DOI:** 10.1016/j.lana.2026.101452

**Published:** 2026-03-17

**Authors:** Kehinde O. Ogunyemi, Affan T. Shaikh, Adnan Bashir, Brian E. Dixon, Dee Warmath, Morgan Toth, Julian Salim, Virgil K. Lokossou, Fitsum Teferi, Caroline Baer, Jayson Brown, Lionel S. Sogbossi, Tanzeel Zohra, Daniel Tom-Aba, Andrew S. Awori, Ahmed Haji Said, Yewen Chen, Simon Antara, Wondwossen A. Gebreyes, Amy K. Winter, Hammad Ali, Neil Squires, Senait Kebede, Rana J. Asghar, Stella Chungong, Wondimagegnehu Alemu, Melchior A. Aïssi, Juliet N. Sekandi, Tadesse Wuhib, Chima Ohuabunwo, Ye Shen, Scott J.N. McNabb

**Affiliations:** a University of Georgia, College of Public Health, Department of Epidemiology and Biostatistics, GA, USA; b Emory University, Rollins School of Public Health, Hubert Department of Global Health, GA, USA; c Health Information Systems Program, Islamabad, Pakistan; d Indiana University, Fairbanks School of Public Health, Department of Epidemiology, IN, USA; e Center for Biomedical Informatics, Regenstrief Institute, IN, USA; f University of Georgia, College of Family and Consumer Sciences, Department of Financial Planning, GA, USA; g Public Health Practice, LLC, FL, USA; h University of Washington, Department of Global Health, Washington, USA; i West African Health Organization, Bobo Dioulasso, Burkina Faso; j Emory Saint Joseph's Hospital, GA, USA; k US Centers for Disease Control and Prevention, Malaria Branch, GA, USA; l AI for Public Health & Informatics, Inc, GA, USA; m African Field Epidemiology Network, Kampala, Uganda; n The Ohio State University, The Global One Health Initiative, OH, USA; o EpiCare Consulting, GA, USA; p Brown University, RI, USA; q International Association of National Public Health Institutes, Brussels, Belgium; r Emory University, Center for Studies of Human Health, GA, USA; s Global Health Strategists and Implementers, Islamabad, Pakistan; t World Health Organization, Health Emergencies Program, Geneva, Switzerland; u University of Georgia, Global Health Institute, GA, USA; v US Centers for Disease Control and Prevention, Center for Global Health, GA, USA; w Morehouse School of Medicine, David Satcher Global Health Equity Institute, GA, USA

**Keywords:** Emerging infectious diseases, One Health, Public health surveillance, Public health emergency management, Artificial intelligence, Global health security

## Abstract

An electronic public health surveillance (e-PHS) embracing One Health and participatory approaches will collect and analyze data at the human-animal-environment interface to enhance real-time information for the prevention and control of public health emergencies (PHE) such as infectious disease outbreaks. Yet full implementation is suboptimal worldwide. Leveraging the capabilities of emerging digital technologies legally and ethically, we described the scope, added benefits, and applicability of a novel cloud-based, artificial intelligence-enabled One Health Integrated Disease Surveillance and Response health information system (AI-OneHIS) data infrastructure for modernizing the existing traditional PHS models. This multifaceted innovation will ensure faster data capture, seamless interoperability of fragmented HIS, and precise decision support, while preserving their structures, functionalities, and capabilities for routine operations and data sovereignty. This should enable the prevention, timely detection, and effective response to PHE for improved health outcomes if implemented with fidelity on a strong governance-collaboration-informatics-analytics framework.

## Introduction

Electronic public health surveillance (e-PHS) enabled through ethical artificial intelligence (AI) and One Health-based Integrated Disease Surveillance and Response (IDSR) is the future of global health security to prevent, detect, and respond to emerging and endemic public health hazards ranging from infectious diseases to natural disasters.^1^, ^2^, ^3^, ^4^, ^5^ The interconnectedness and complexity of interactions at the human-animal-environment interface demand these high-yield evidence-based approaches.^6^, ^7^, ^8^, ^9^, ^10^, ^11^, ^12^, ^13^, ^14^, ^15^, ^16^, ^17^ One Health (i.e., the collaborative efforts of multiple disciplines, working locally, nationally, and globally to attain optimal health for people, animal, and the environment) and ethical AI (i.e., AI systems that adhere to well-defined ethical guidelines on fairness, non-discrimination, transparency, accountability, and privacy) are crucial to address public health risks from climate change, pathogen spillover, and intentional pathogen release, as shaped by the vulnerabilities and capacities of health systems.^5^, ^6^, ^7^, ^8^, ^9^, ^10^, ^11^, ^12^^,^^14^, ^15^, ^16^

In addition, external factors like social (e.g., travel or travel bans), economic (e.g., trade or trade restrictions), and technological (e.g., social media infodemics or social media fact checking) can shape or be shaped by public health emergencies (PHE).^18^, ^19^, ^20^, ^21^, ^22^, ^23^, ^24^, ^25^ These complex interactions underscore the need for a robust public health ecosystem with modern tools and technologies. Integrating One Health and e-PHS leveraging ethical AI, defines the public health ecosystem while implementing a whole-of-society, multidisciplinary, and comprehensive strategy for PHE management rooted in global health frameworks (e.g., International Health Regulations [IHR 2005]). It leverages digital technology and offers the next game changing opportunity to ensure a better protected and safer planetary ecosystem by helping to reduce the burden of zoonotic diseases (i.e., infectious diseases that can be transmitted from vertebrate animals to humans).^9^, ^10^, ^11^, ^12^^,^^26^, ^27^, ^28^, ^29^, ^30^ Evidence shows that when a traditional PHS model involving case-, event-, and syndromic-based surveillance is combined with innovative PHS that supports community, genomic, and crowdsourced participation, the time to detect an outbreak can be reduced by more than 50%.^27^

With an interconnected public health ecosystem backed up with sustained multisectoral, political, technical, and economic commitment that embraces equitable policies and standards, plus all-hazards strategies and actions, the global health community has a window of opportunity now to maximize the potentials of ethical AI and other emerging digital technologies (e.g., cloud computing, blockchain).^15^^,^^29^^,^^31^ This can be achieved through the triangulation of data on outbreaks emergence in the animal population and changes in climatic conditions (e.g., temperature, precipitation) to generate timely actionable insights. These insights can provide early warnings for potential climate-sensitive zoonotic diseases outbreaks (e.g., influenza, Ebola) in the human population.^8^, ^9^, ^10^, ^11^, ^12^

The COVID-19 pandemic exposed the ill-prepared state of many health systems, including the United States (U.S.). Challenges include cumbersome and delayed data entry through manual or siloed electronic health records (EHRs),^32^^,^^33^ non-interoperability and synchronicity of health information systems (HIS),^34^^,^^35^ prolonged and unsecured data exchanges between healthcare and public health data infrastructures,^34^^,^^35^ and a demand-supply mismatch of information and communication technology (ICT)-savvy health workforce.^32^^,^^33^^,^^36^^,^^37^

For example, in the U.S., evidence showed that during the COVID-19 pandemic, up to 70% of healthcare institutions reported cases to states, tribes, localities, and territories (STLT) and the Centers for Disease Control and Prevention (CDC) through a fax-based system.^34^ In a CDC report, it was also revealed that about 80% of epidemiologists experienced additional work burden from data cleaning due to non-interoperability issues.^35^ These findings aligned with national professional and government recommendations.^17^^,^^33^^,^^38^ These emphasize the need for increased investment in public health core data, surveillance, and intelligence capabilities through legislation, research, education, capacity building, collaboration, and resource mobilization for faster data access, robust analysis, and effective decision-making.

These PHS data infrastructure problems are similar to what have been reported in other global settings like those in Africa (e.g., Zambia, Nigeria) and the Latin America/Caribbean (e.g., Brazil, Ecuador).^39^ Most of these settings have consistently been faced with limited data access and quality, poor internet connectivity, and incoherent digital policy and regulatory implementation issues, in part, driven by weak health systems, ineffective digital governance, and budgetary constraints.^14^^,^^21^^,^^39^ For instance, recent evidence suggests that the case reporting rate for COVID-19 in low- and middle-income countries (LMICs) was as low as 5.4%–14.3%.^39^^,^^40^ The pandemic revealed how most of the world's PHS data infrastructure were unable to match up with data needs for reliable burden assessment, consistent evidence-based risk communication, and urgent policy prioritization and evaluation.

Importantly, having a reimagined national health (both healthcare and public health) ecosystem with modernized data infrastructure and functions to better meet current and future needs, is a matter of global health and national security, and a key effort to sustainable economic and human development.^17^ While some progress has been recorded with ongoing initiatives and government efforts (e.g., eIDSR, automated case reporting),^3^^,^^41^^,^^42^ the operational information needed to demonstrate the potential added benefits, performance, and scalability of an interconnected public health ecosystem using e-PHS and One Health approaches for PHE management, remains a major challenge.^17^^,^^33^^,^^38^

In this Health Policy, we argue that complementing existing PHS models with a cloud-based, AI-enabled One Health IDSR HIS (AI-OneHIS) data infrastructure in an interconnected public health ecosystem is crucial to integrate and harmonize fragmented HIS for maximized PHS benefits and minimal risks.

## Main

### Modernizing public health surveillance for global health security

Global health security (GHS), defined as “the activities required, both proactive and reactive, to minimize the danger and impact of acute public health events that endanger people's health across geographical regions and international boundaries”,^43^ is now at an inflection point. Modernizing PHS for GHS with the full implementation of One Health and participatory approaches leveraging AI, is urgent more than ever for universal health coverage, national security, economic growth, and sustainable development.^3^^,^^5^^,^^27^^,^^28^^,^^31^ This is crucial, particularly, considering the unprecedented realities of post-pandemic preparedness, climate change, and digital and AI maturity that are now shaping health, education, and finance decision-making at the individual, population, community, and organizational levels worldwide.^8^^,^^13^^,^^15^ Notably, the inextricable interdependence of a modernized PHS and GHS in these contexts are multidimensional. Regarding post-pandemic preparedness, first, a modernized PHS will help bring data into action to protect the health and livelihoods of people worldwide against infectious disease threats. Second, having a well-protected health and livelihoods of people will help ensure health authorities are trusted and timely health data become available when needed to inform meaningful policy interventions and investments. Third, having meaningful policy interventions and investments will help sustain the protection of the health, livelihoods, and data of people.

Apart from the increased risk of novel infectious pathogens emergence and expansion of emerging ones in the human, animal, and plant ecological niches, climate change from excessive heat or cold could significantly erode some of the hard-won gains in GHS in other mutually reinforcing ways.^8^^,^^9^ First, is the increased risks of cardiometabolic diseases (e.g. hypertension, diabetes), low work productivity, and nutrients loss within the human, animal, and plant populations, respectively, through physiological stress.^8^ Second, cardiometabolic diseases may in turn contribute to the weakening of populations immunity and reduction in their access to essential foods (e.g., meats, vegetables), resulting in higher susceptibility to infectious diseases in the short-term and associated poor health outcome in the long-term. Third, is the increased risk of flooding or drought that could undermine case reporting, water, sanitation and hygiene (WASH) and infection prevention and control (IPC) infrastructures that are vital for mounting optimal infectious disease outbreaks response.

In both situations, when AI is leveraged, it offers unique opportunities to improve the linkage of: 1) health data to public health action (e.g., faster outbreak and climate event detection by analyzing data for early warnings, reliable risk communication and community engagement by countering outbreak misinformation and curating resources for climate change mitigation), and 2) public health action to health-promoting behaviors (e.g., high outbreak or climate event risk perception by personalizing outbreak and climate information alerts, case reporting by providing alternative sources for outbreak and climate event case detection through mobile phones). These suggests that sustainable investment in a PHS data infrastructure modernization leveraging AI can only be successful if developed in tandem with climate change mitigation interventions on awareness, prediction, and social infrastructure. But achieving these gains requires that health systems at the national, regional, and global levels are guided by a strong governance-collaboration-informatics-analytics framework. This is necessary to transform a PHS model from an “incomplete and fragmented state without a roof of current transnational policy and regulatory instruments” to a “complete, organized, and integrated state with a roof and sequential workflow processes”, as argued by McNabb and colleagues.^44^

#### The building blocks of a modernized PHS model

##### Governance: e-PHS, digital health, and AI strategic action over policy proliferation

The national, regional, and global e-PHS, digital health, and AI (PDA) landscapes are fast maturing, but are unfortunately becoming increasingly fragmented and failing to scale, making governance the weakest link in this digital chain of PHS modernization.^3^^,^^15^^,^^17^^,^^31^^,^^33^^,^^38^^,^^44^ Recognizing that ethics and human rights must drive the co-creation and use of these advancements, over the past five years, we have seen several soft policies (e.g., guidelines: WHO Global Initiative on AI for Health, U.S. Food and Drug Administration [FDA] AI Framework, African Union Development Agency [AUDA-NEPAD] Digital Transformation Strategy) and a few hard policies (e.g., regulation: European Union [EU] AI Act) dominating academic, political, civil society, and the private sector spaces.^45^, ^46^, ^47^

While these policies are necessary they are not sufficient to achieve the desired goals if they are not complemented with strategic action targeting the provision of: 1) *harmonized digital policies* through cross–functional activities (e.g., ethics and regulatory enforcement, implementation facilitation); 2) *enabling research and development environment* through improved local human talent harnessing (e.g., hackathon challenge, digital scholarship), digital inclusion (e.g., internet access, digital literacy), and local digital production (school education, ICT training); and 3) *predictable and sustainable digital investment* through locally-resourced and internationally-supported funding (e.g., digital fund, social entrepreneurship). More so, practical governance mechanisms must include the establishment of a cross-sectoral ethics review board with a structure that is inclusive of representatives from ministries of health (MoH), national public health institutes (NPHIs), national ICT authority, legal departments, academic institutions, patients advisory groups, and civil societies, providing operations in the areas of digital health technologies design and development, effectiveness and safety evaluation, and deployment and dissemination.^31^^,^^45^ Specific operations of this board should include collaborative stakeholder engagement, regulatory and ethical framework adherence, ethical AI development (design and development oversights), outcome measures adequacy, study design and statistical plan appropriateness, pre-pilot test registration (effectiveness and safety evaluation), and continuous effectiveness and safety reporting, learning and quality improvement monitoring, implementation and operational research guidance (deployment and dissemination).

Additionally, data sovereignty and algorithmic fairness should be ensured within a federated cloud architecture, with the role of a cross-sectoral data governance board (with representatives as above) specified in the pre-deployment and post-deployment phases of PDA implementation.^31^^,^^45^ For example, pre-deployment oversights should encompass regulatory consensus and compliance, localized data access and storage, and algorithmic AI debiasing, while post-deployment oversights should involve continuous data standards and processing monitoring, cloud standards and operations maintenance, and deployment accountability and penalties enforcement. Practically, the data governance board will oversee AI algorithm approval, monitor AI model and systems performance for fairness, non-discrimination, transparency, accountability, and privacy across regions and populations, and enforces data-access controls. And within the same cloud architecture, data sovereignty must be ensured by storing and processing identifiable data within country-owned infrastructure (i.e., data warehouse controlled by MoH and NPHIs), while only de-identified or model parameters are exchanged across nodes. Algorithmic fairness can be operationalized using periodic bias audits, transparent model documentation (e.g., model cards), and human interfacing (e.g., decision logs), and health-system-defined guardrails that restrict autonomous decision-making. Notably, health systems that can center these recommendations on equity, transparency, and accountability principles would have provided an enabling environment for meaningful collaborations.

##### Collaboration: transdisciplinary over multidisciplinary

Like the current policy positioning for PDA governance,^31^^,^^45^, ^46^, ^47^, ^48^, ^49^, ^50^, ^51^, ^52^, ^53^, ^54^, ^55^, ^56^, ^57^ evidence from the field suggests that collaborations are also rapidly increasing and observed to be multidisciplinary in structure and operations. However, health institutions, particularly those in LMICs need to seek collaborations more inwardly than outwardly from the transdisciplinary lens for transformational impact on health. This type of collaboration provides the necessary foundation for mixed evidence generation, co-designing, agile infrastructural support, seamless data sharing agreements, bidirectional technology transfer, and easier competing interest resolution. Importantly, health institutions and countries need to strategically identify, engage, expand, and nurture stakeholders (ICT experts, practitioners, policymakers, populations) locally and form coalitions for mixed and shared expertise on common PHS informatics and analytics activities.

##### Informatics: alignment over replacement

At the core of a modernized PHS model, informatics plays a central role in maximizing the use of data for public health action.^15^^,^^31^^,^^44^ This is achieved through effective and ethical design, development, pilot testing, deployment, and dissemination of PDA solutions. This involves ensuring that these solutions are adequately prioritized, resourced, aligned, and used with existing digital infrastructure to meet the unique contexts and needs of health institutions and countries. But beyond their effectiveness, consensus-driven and transparent efforts must be pursued to ensure that they are designed and developed with robust and resilient technologies using common data and technology standards. These include evidence-based cross-cutting and design-specific standards (i.e., data element, data exchange, data security, data infrastructure cloud hosting, and monitoring and evaluation) that adhere to ethical guidelines (fairness, non-discrimination, transparency, accountability, and privacy), and global data principles (e.g., FAIR: findability, accessibility, interoperability, and reusability, and SMART: standards-based, machine-readable, adaptive, requirements-based, and testable) to enhance the interoperability of PHS data infrastructure for actionable intelligence.^15^^,^^45^^,^^48^^,^^49^

##### Analytics: integrated intelligence over siloed dashboard

There is a mismatch between the scale and speed of data needs and PHS for public health action, as demonstrated during the COVID-19 pandemic.^15^^,^^28^^,^^31^ Health institutions and countries must ensure that necessary tools, processes, people, and provisions are harnessed and deployed to generate real-time, precise actionable intelligence using PHS model integrated with flexible analytics options other than rigid, siloed dashboards. This should be enabled through the development of an interoperable PHS data infrastructure that allows for comprehensive analysis of health data using multiple data sources (e.g., One Health, crowdsourced), mixed expertise (e.g., academicians, policymakers), mixed epidemiological models (e.g., statistical, machine learning), and real-world model parameter selection, assumptions making, policy scenarios setting, and prospective model estimates correction, as data become available and context changes. An example of a modernized PHS model built on a strong governance-collaboration-informatics-analytics framework can be pictured from a proposed innovation, as presented below.

### A case study of the U.S.

#### PHS modernization context and needs

In the U.S., gathering and interpreting PHS data is a shared effort among thousands of units at STLT levels. Numerous challenges persist as shown during the COVID-19 pandemic. Commenting to *Politico*, a former CDC director stated “there are real structural problems in states that don't have the data. There are structural problems in CDC's ability to get data from states and health facilities, the challenge is big, and it is not easy to solve. There are no quick fixes here.”^50^ Similar views were also shared by five former CDC directors.^51^ These challenges are complex and multidimensional. The country's public health system is constrained with +120 siloed, PHS systems and +173 health IT vendors who supply certified EHRs products to +4500 hospitals; +20 million paper records generated per site per year; and +3000 federal and STLT partners, reflecting a substantial PHS collaboration, informatics, and analytics gap.^41^ While, laudable investments have been made to close this gap (e.g., the 2019–2024 CDC Data Modernization Initiative (DMI) and Public Health Data Strategy [PHDS]) to ensure critical data are exchanged efficiently and securely across healthcare and public health for resilient emergency response-ready public health systems, several PHS data, technology, policy, and administrative needs remain unmet. By advancing the CDC's North Star Architecture (NSA), AI and Machine Learning, Vital Statistics Modernization Tools, Open Technology, and HL7 fast healthcare interoperability resources (FHIR) underlying these efforts,^41^^,^^42^ this Health Policy proposes a new way forward with a novel multifaceted innovation. This innovation has the potential to achieve the unmet needs of providing accurate, sensitive, specific, and timely PHS data, information, and messages where they are needed and when they are needed, and the action steps needed for its implementation and potential scale up. This involves powering the public health ecosystem with an AI-OneHIS data infrastructure and deploying it in alignment with the existing healthcare ecosystem, which has a completely different workflow process.

#### Traditional PHS model

Regarding governance, the main challenge of the traditional PHS model in the U.S. is that there are many proprietary EHRs and laboratory information management systems (LIMS) that operate in siloes.^29^^,^^32^^,^^33^^,^^38^ This inefficiency could be attributed to the lack of cohesive laws, regulations, and policies needed to minimize case reporting burden (multiple data entry, incomplete and non-interoperable data cleaning, additional trainings for different PHS databases), as well as to mitigate risks (privacy, confidentiality, security).^32^^,^^33^^,^^38^ Concerning informatics, some EHRs are web-based while others are fragmented programs running on standalone computers or local networks. They vary in design, coding language, and coding platforms.^32^, ^33^, ^34^^,^^41^ Integrating and harmonizing these various systems and making them interoperable to abstract healthcare data to feed critical public health data to appropriate public health stations (county, state, federal) has been challenging and resource-intensive. In addition, not all reportable conditions, including veterinary and pharmacy reportable conditions (e.g., avian influenza, vaccine adverse event) are linked within this traditional model.^32^, ^33^, ^34^

Likewise, there is limited capability of the data infrastructure at the STLT and CDC to abstract other core (animal health, environmental health, pharmacies, crowd-sourced [e.g., mobility, social media, public trust]) data for PHE management, in addition to its routine core data sources, including case, laboratory (i.e., molecular, serologic, and genomic tests), emergency department, vital statistics, immunization, healthcare capacity and utilization, and wastewater data.^33^^,^^38^^,^^42^ The cumulative effects of these deficiencies in PHS governance and informatics then, in turn, challenge the generation of timely and accurate analytics for evidence-informed public health action (e.g., outbreak investigation and contact tracing, testing, treatment) and optimal resource allocation.

### Modernized PHS model

#### Hypothesis

**Fig. 1:**
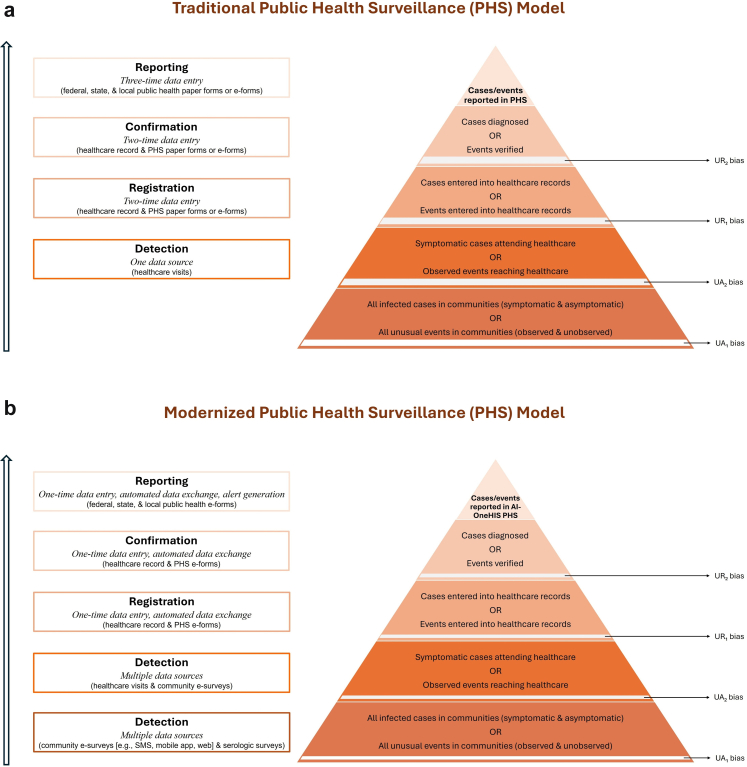
Comparison of the traditional and modernized public health surveillance (PHS) models by core activities, data infrastructure functionalities, data collection methods, and underestimation bias. AI-OneHIS denotes a cloud-based, AI-enabled One Health Integrated Disease Surveillance and Response health information system. Underestimation bias is calculated as: underreporting (UR) bias + underascertainment (UA) bias, where UA bias = UA_1_ bias + UA_2_ bias and UR bias = UR_1_ bias + UR_2_ bias. Calculations for UA_1_ bias = number of all infected cases or unusual events in the total population—number of symptomatic cases or observed events detected in communities, UA_2_ bias = number of all infected cases or unusual events detected in communities—number of symptomatic cases or observed events detected in healthcare, UR_1_ bias = number of cases or events detected in healthcare—number of cases or events registered, and UR_2_ bias = number of cases or events registered—number of cases or events confirmed. Modernized PHS model (Fig. 1b) should have lesser underestimation bias due to community participation and reduced data entry/exchange errors compared to traditional PHS model^2^^,^^52^ (Fig. 1a).

We hypothesize that a modernized PHS model with an AI-OneHIS data infrastructure, supporting *one-time data entry, secure automated data exchange, and automated alert and intelligence generation* functionalities for seamless integration, harmonization, and abstraction of routine human health data and other *One Health* (animal health and environmental health, pharmacy and crowdsourced human health) data will improve the timeliness and precision of outbreak detection, response, and monitoring and evaluation compared to the traditional PHS model (Fig. 1).

#### Development

In designing this model, we leveraged the field experience of all authors in this study, who have expertise in PHE preparedness and response across academia, government, and the private sector. We then synthesized knowledge from the above-described U.S. PHS model analysis to inform the selection of our modernized PHS model components. Prioritization of the selected components were informed by the competitive advantages they offer compared to counterparts, in addition to their feasibility for widespread scale up. Through an iterative process, including internal reviews (discussion among authors) and external reviews (peer review invitation to colleagues via email and feedback from PHS-related conference presentations), we refined this PHS model until a consensus for its final design was reached.

#### Innovation and workflow process

We demonstrate a comprehensive, modernized PHS model to capture and analyze state-mandated reportable public health conditions from hospitals, laboratory system, border control health authorities, animal clinics, environmental departments, pharmacies, and the public within a new public health ecosystem to provide real-time, automated reporting to public health authorities at all levels. This PHS model offers three distinct interconnected innovations, providing core functionalities at the human-animal-environment interface namely:

1. **Faster data capture** from multiple sources with one-time data entry.
2. **Seamless interoperability** of data systems with secure automated data exchange.
3. **Precise decision support** for public health workers with automated alert and intelligence generation.

**Fig. 2:**
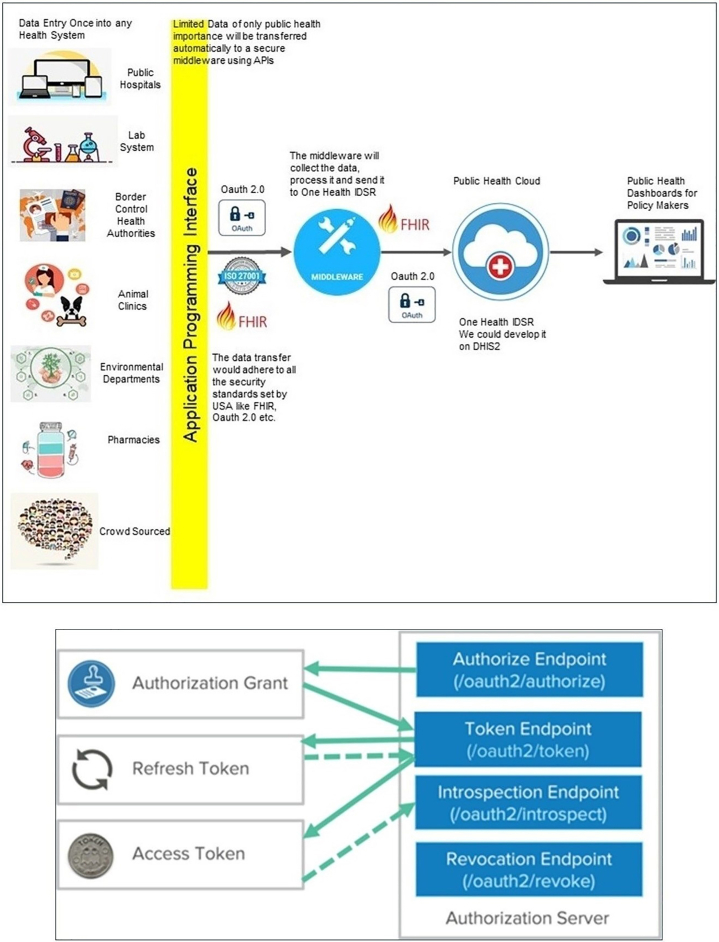
Upper schematic shows the workflow process of a modernized public health surveillance model using a cloud-based, AI-enabled One Health Integrated Disease Surveillance and Response health information system (AI-OneHIS) data infrastructure. AI-OneHIS is shown to have data capture (*one-time data entry*), interoperability (*automateddata exchange*), and decision support (automated alert and intelligence generation) innovations and core functionalities with capabilities for nowcasting and forecasting in the event of a public health emergency (e.g., infectious disease outbreaks). Lower schematic shows authorization grant, refresh token, and access token that interact with the authorization server. On the right, the server provides authorize, token, introspection, and revocation endpoints for obtaining authorization, exchanging grants for tokens, validating tokens, and invalidating them, respectively. Solid arrows show the direct, standard, expected flows in OAuth2. Dotted arrows indicate optional, indirect, or secondary interactions.

This model is enhanced with nowcasting and forecasting capabilities using epidemiologic models that triangulate core health data and non-health (e.g., mobility, economic) data to understand infectious diseases transmission dynamics, as shaped by human movement, social protection policy, and risk communication.^18^^,^^19^ This is essential to rapidly, effectively, and efficiently respond to outbreaks, as demonstrated during the 2022 U.S. mpox outbreak and the 2019–2023 COVID-19 pandemic.^18^^,^^53^^,^^54^ AI-OneHIS data infrastructure aims to serve as a national public health HIS based on real-world, real-time sharing of critical and reportable data. This will be achieved using the middleware platform, application programming interface (API), and ethical generative AI that make relevant data across multiple sectors interoperable, readable, and useable.^55^, ^56^, ^57^, ^58^, ^59^ A middleware platform can make disparate legacy and emerging EHRs and technologies interoperable, enabling real-time access via a unified interface like in finance and retail industries,^60^ as demonstrated in Fig. 2.

#### Alignment with existing data infrastructure

Our proposed innovation aligns with the CDC's NSA (i.e., “a blueprint and core component of the CDC approach for making public health data work better”) that aims to test a potentially scalable and flexible data infrastructure that is standardized and secure within a cloud environment. This will be achieved by prioritizing collaboration and transparency, to ensure that all stakeholders can ethically access and utilize the data effectively.^41^^,^^42^ It leverages nine automated components with human in the loop, as described in Table 1.

**Table 1:** Detailed workflow process of a modernized public health surveillance model using a cloud-based, AI-enabled One Health Integrated Disease Surveillance and Response health information system (AI-OneHIS) data infrastructure.

Component	Overview	Implementation	Example
**Data capture**			
*Data-entry Once*	The “data-entry once” approach ensures that data are entered only one time by any user, after which the data are automatically distributed simultaneously into all relevant systems, including electronic health records (EHRs), laboratory information management systems (LIMS), veterinary record systems (VRS), environmental record systems (ERS), and pharmacy record systems (PRS). Data containing public health reportable conditions are determined and then distributed into the public health ecosystem called the One Health Electronic Integrated Disease Surveillance and Response health information system (One Health eIDSR HIS).	**Platform**: A secure web platform will be developed using React JavaScript, known for creating fast, responsive, and user-friendly applications compatible with various devices (smartphones, tablets, iPads, laptops, desktops). **Process**: Users (e.g., physicians, nurses, laboratorians, veterinarians, environmentalists, pharmacists, the public) enter data into the React-based platform. **Distribution**: The data are securely and automatically distributed to EHRs, LIMS, VRS, ERS, and PRS and then into the One Health eIDSR HIS (using ethical generative AI algorithms).	A nurse enters a patient's vital signs and initial diagnosis into the system during a hospital visit. This information is automatically and simultaneously shared with the hospital's EHRs, the laboratory (lab)'s LIMS for any test requests, and the One Health eIDSR HIS for public health reporting.
**Interoperability**			
*Middleware Platform*	The middleware platform acts as a bridge, ensuring data entered once are concurrently and simultaneously reported into all relevant systems.	**Development**: The middleware will be developed in Spring Boot, a robust Java-based framework known for its flexible configurations and reliable batch processing. **Security**: Data transfer will adhere to ISO 27001 standards, using encryption and OAuth 2.0 authentication with TLS cryptographic algorithms. **Functionality**: The middleware ensures that data relevant to public health reporting are transferred in real-time into the One Health eIDSR HIS.	A veterinary laboratory records a test result for a zoonotic disease. The middleware ensures this result is securely transferred to the VRS, LIMS, and the One Health eIDSR HIS simultaneously.
*Application Programming Interface (API)*	The API facilitates communication between software applications, enabling seamless data exchange.	**Integration**: API will be integrated to allow different systems (e.g., EHRs, LIMS, VRS, ERS, PRS) to exchange data effortlessly. **Accessibility**: API ensure that data can be shared across different platforms and organizations, enhancing interoperability.	When a new case of a reportable disease is entered into the system, an API sends this information to the state health department's PHS system, ensuring timely reporting and response.
*OAuth 2.0 Authentication*	The OAuth 2.0 is an authentication protocol that allows secure authorization without sharing passwords.	**Tokens**: OAuth uses authorization tokens to verify identities, ensuring secure data exchanges between systems. **Protocol**: This protocol ensures that users approve one application to interact with another on their behalf, without revealing passwords.	A public health official accessing the One Health eIDSR HIS to review reported case data uses OAuth 2.0 authentication to log in, ensuring their credentials remain secure while accessing sensitive information.
*Robotic Process Automation (RPA)*	The RPA automates repetitive tasks, such as data entry, by using software robots or “bots”.	**Development**: RPA will be developed using UiPath to automate data entry into EHRs, LIMS, VRS, ERS, and PRS. **Functionality**: RPA observes and replicates user actions in an application's GUI, ensuring tasks are performed accurately and efficiently without the need for API in proprietary systems.	An RPA bot logs into a hospital's EHRs system, enters patient information and initial diagnosis, and then logs into the LIMS to input the corresponding lab. Test requests. This automation reduces manual workload and minimizes errors.
*Electronic Integrated disease Surveillance and Response (eIDSR)*	The One Health eIDSR HIS will be built on DHIS2, an open-source platform widely used for health data reporting and analyses.	**Platform**: DHIS2, used in over 70 countries, provides a flexible and integrated platform for data reporting, analysis, and dissemination **Ethical AI**: The system will leverage ethical AI to detect, confirm, analyze, and report public health data, enhancing PHS capabilities.	The system detects an unusual spike in respiratory illness cases in a particular region. Ethical AI algorithms analyze the data and alert public health officials, prompting further investigation and a potential response to a disease outbreak.
*Fast Healthcare Interoperability Resources (FHIR)*	The FHIR is a standard for electronic exchange of healthcare information, promoting interoperability.	**Framework**: FHIR provides modular components (resources) that can be assembled to resolve various healthcare challenges. **Web Standards**: FHIR supports web-based standards such as XML, JSON, HTTP, and OAuth, facilitating efficient data exchange and retrieval.	Not applicable.
**Decision support**			
*Ethical Artificial Intelligence (AI) Module*	The ethical AI module enhances the One Health eIDSR HIS by analyzing data to identify all public health reportable conditions.	**Training**: The ethical AI module will be trained using historic data sets, such as the National Notifiable Diseases Surveillance System (NNDSS). **Alert system**: Upon detecting data of public health importance, the system will generate alerts through various channels (e.g., emails, SMS, automated calls).	Ethical AI algorithms identify patterns indicating a possible outbreak of a foodborne illness. The system sends automated alerts to local health departments, enabling a swift investigation and response.
*Ethical Generative AI System*	The ethical generative AI system can enhance the public health surveillance (PHS) model by developing deep learning algorithms around case definitions of diseases, aiding in recognition of reportable conditions, and providing information (e.g., line lists, epi curves) to public health officials at all levels.	**Case definitions**: Ethical generative AI system will be trained on extensive datasets to develop and refine case definitions of various diseases. This system will provide analytic presentations (e.g. line lists, epi curves) to public health officials. **Interactive system**: Public health officials can interact with the PHS model to get real-time insights for responses based on the latest guidelines and research. **Continuous learning**: The ethical generative AI system will continuously learn from new data, improving its accuracy and the relevance of its recommendations over time.	A healthcare provider enters a patient's symptoms into the system. The ethical generative AI system provides case reports and information about potential public health threats based on real-time data analysis.

AI-OneHIS is described with one-time data entry, automated data exchange, and automated alert and intelligence generation core functionalities and the capabilities for nowcasting and forecasting in the event of a public health emergency (e.g., infectious disease outbreak).

#### PHS models comparison

To demonstrate how our proposed modernized PHS model can transform PHE preparedness and response, building on existing health systems workflow, we highlight its capabilities by components, potential opportunities, and relative advantages across essential data workflow processes. These included 1) data source, 2) data collection and collation, and 3) data analysis and dissemination, in addition to their overarching approach, as shown in Table 2.

**Table 2:** Comparison of modernized and traditional public health surveillance models in the United States.^41^^,^^42^

Data workflow	Traditional PHS model	Modernized PHS model	US CDC's DMI/PHDS
Overarching approach	**Component**: • North Star Architecture **Gap**: • Focuses only on human healthcare data • Partially cloud-based public health agency surveillance infrastructure • Partially AI-enabled PHS model **Advantage**: • Effective data access, management, and analysis for public health action	**Component**: • North Star Architecture **Opportunity**: • One Health and other health data • Fully cloud-based public health agency surveillance infrastructure • Fully AI-enabled PHS model **Relative advantage**: • More effective data access, management, and analysis for public health action	**Mission**: Robust ability to detect and monitor public health threats through near real-time and secure data exchanges; investigate and respond; be response-ready; and inform and disseminate
**Data capture** Data source	**Component**: • Routine core human health data (case, laboratory, emergency department, vital statistics, immunization, and healthcare capacity and utilization) • New data (wastewater) **Gap**: • Limited data access **Advantage**: • Data access for actionable insights	**Component**: • Routine core human health data • New data (animal health, environmental health, pharmacy, and crowdsourced) **Opportunity**: • Robust data access **Relative Advantage**: • Data access for real-world actionable insights	**Goal 1**: Strengthen the core of public health data
**Interoperability** Data collection and collation	**Component**: *Data collection* Human healthcare system (hospital and laboratory) • EHRs and LIMS Public health system STLT • eICR • Cloud-like NEDSS Base System CDC • NNDSS *Data collation* • NEDSS Base System and NNDSS from eCR or PHDC in EHRs and LIMS using HL7 via HIE platform **Gap**: • Error-prone manual data exchange between EHR and LIMS leading to uncoordinated patient care or missed cases • Complex, non-streamlined, and security risk-prone data exchange due to limited interoperability of HIE **Advantage**: • Access to somewhat timely, complete, valid, and secure data for public health action	**Component**: *Data collection* One Health system (hospital and laboratory, animal clinic, environment health department) • EHRs and LIMS, VRS, ERS + Data entry once using RPA Other health system (border control, community) • PRS • EDN System • Crowdsourced Public health system STLT • eCR • Cloud-based One Health eIDSR HIS CDC • NNDSS *Data collation* • One Health eIDSR HIS and NNDSS from eCR in EHRs and LIMS using API, FHIR, OAuth 2.0 authentication, Ethical AI module via HIE + Middleware platform **Opportunity**: • Automated data exchange • Simplified, streamlined, and secure data exchange **Relative Advantage**: • Access to more timely, complete, valid, and secure data for public health action	**Goal 4**: Advance more open and interoperable public health data
**Decision support** Data analysis and dissemination	**Component**: • EDAV **Gap**: • Manual outbreak alerting and limited public health insights generation **Advantage**: • Near real-time data insights for timely and effective public health action	**Component**: • EDAV + Ethical AI module and Generative AI **Opportunity**: • Automated outbreak alerting and AI-enabled public health intelligence generation **Relative Advantage**: • Real-time and robust data intelligence for more timely and effective public health action	**Goal 2**: Accelerate access to analytic and automated solutions to support public health investigations and advance health equity **Goal 3**: Visualize and share actionable insights to inform public health actions

US, United States; PHS, Public Health Surveillance; CDC, Centers for Disease Control and Prevention; DMI, Data Modernization Initiative; PHDS, Public Health Data Strategy; AI, Artificial Intelligence; EHRs, Electronic Health Records; LIMS, Laboratory Information Management Systems; VRS, Veterinary Record Systems; ERS, Environmental Record System; RPA, Robotic Process Automation; STLT, States, Tribes, Localities, and Territories; eICR, Electronic Initial Case Report; NEDSS, National Electronic Disease Surveillance System; NNDSS, National Notifiable Diseases Surveillance System; eCR, Electronic Case Report; PHDC, Public Health Document Container; HL7, Health Level Seven; HIE, Health Information Exchange; PRS, Pharmacy Record Systems; EDN, Electronic Disease Notification; eIDSR, Electronic Integrated Disease Surveillance and Response; HIS, Health Information System; API, Application Programming Interface; FHIR, Fast Healthcare Interoperability Resources; EDAV, Enterprise Data, Analytics, and Visualization.

### Policy and practice implications

The policy and practice implications of this modernized PHS model are categorized in four ways, encompassing potential technical, administrative, social, and research benefits.

Regarding technical benefits, this model can support robust risk assessment for situational awareness and decision-making through nowcasting/forecasting to prevent, detect, and respond to known or new PHE at the human-animal-environment interface (i.e., improved representativeness and data quality). This can be achieved using a mixed-methods or model-based approach in line with global best practices such as the Joint Risk Assessment Operational Tool (JRA OT) and the Strategic Tool for Assessing Risks (STAR).^61^^,^^62^ Additionally, given the potential capability of this modernized PHS model to capture data from multiple sources such as the public (e.g., infected, symptomatic undiagnosed) through community participatory surveillance (e.g., SMS, web surveys) that are rarely ascertained in the traditional PHS model,^63^, ^64^, ^65^ public health officials and policymakers will better detect and estimate the true burden of the PHE. This will provide real-world evidence for effective resource mobilization and response, resulting in improved validity and sensitivity of this PHS model. As a broader impact, this will contribute to the reduction of morbidity and mortality and associated socioeconomic losses through faster case reporting, timely outbreak detection, and effective response.^15^^,^^27^^,^^28^

Concerning administrative benefits, this model can improve the productive and allocative efficiencies of the PHE management system at all levels of stakeholders through multisectoral activities (i.e., training, planning, and response) and the efficient use of resources (i.e., human, technical, and financial), respectively. With the assumption that multisectoral collaborations for PHE management will be rooted in open communication, transparency, and ethical and equitable decision-making, such partnerships can be leveraged for the prevention and control of other issues of public health importance (e.g., non-communicable diseases, antimicrobial resistance).

Relating to social benefits, this model can meaningfully contribute to accountability, public trust building, and sustainable partnerships through open data sharing and dissemination best practices such as peer-reviewed publications, public user database availability, and success stories communication.^66^^,^^67^

In terms of research benefits, this model, especially if implemented and scaled up with fidelity, can advance the capability for epidemiologic research under real-world assumptions to generate new hypothesis on the effect of evidence-based or new interventions and policies on public health outcomes before, during, and after a PHE.

### Implementation challenges and opportunities

Despite the potential benefits of this modernized PHS model, there are non-unique and unique challenges and opportunities to data and AI, respectively, to consider for a successful implementation.

#### Data

Like the traditional PHS model, issues around data privacy, confidentiality, and security from unethical data collection and sharing, unauthorized and non-protective data use, and data infrastructure breaches, respectively, may limit the participation of stakeholders (e.g., health sector, non-health sector, and the public).^68^ However, stakeholder participation can be encouraged by mitigating these risks through the enforcement of established national acts and laws on data privacy, confidentiality, and security. Implementers (public health and government) should continuously review data needs, quality, collection, analysis, and dissemination procedures for this PHS model to ensure that they are precise, ethical, legal, and secure. Specifically, PHS implementers should adopt global best data practices (use agreement, de-identification, anonymization, modification) to ensure data integrity and data protection. In addition, although the initial cost of setting up this modernized PHS model may be more expensive compared to the traditional PHS model, its benefits should outweigh the cost and risks in the long term. Hence, this may deter its adoption and institutionalization due to limited resources. Nonetheless, evidence of strong federal technical and financial investments to PHS in several countries suggests a window of opportunity for renewed investments to improve the likelihood of its implementation success.^3^^,^^41^ Lastly, there is also the possibility of underestimation bias due to underreporting (e.g., data entry error, undiagnosed and misdiagnosed) and underascertainment (e.g., from asymptomatic cases, symptomatic cases lacking awareness of case definition) biases.^2^^,^^52^ While this challenge is common to traditional PHS models, the likelihood is less for a modernized PHS model (Fig. 1). This can be addressed through increased multichannel risk communication and decentralized diagnostic interventions, particularly in underserved communities (e.g., rural areas, hard to reach locations).

#### AI

Peculiar to the modernized PHS model, its implementation may be challenged because of the repeated workforce training needs for the ethical AI modules, and further complicated by the existing shortage of skilled ICT personnel.^31^^,^^33^ This challenge can be mitigated by leveraging existing and new collaborations to identify relevant ICT personnel for these training tasks. Notably, due to differences in country laws, priorities, and contexts, the scope and speed of the adoption, and potential scale up of this innovation is unpredictable and may be demanding. Nevertheless, engaging in continued evidence-informed advocacy has the potential to accelerate political commitments and strategic actions through federal, state, and local mandates for its widespread scale up and sustainability.

### Recommendations

Consistent with global best practices,^17^^,^^33^^,^^38^^,^^45^, ^46^, ^47^^,^^69^ we outlined actionable points around policy and standards, and their performance and scalability priorities for modernizing PHS, as discussed below.

#### Policy and standards

We provide 15 actionable policy and standards recommendations, emphasizing PHS policies and funding, capacity building, data interoperability, and data utility across governance, collaboration, informatics, and analytics levels, respectively, for addressing these challenges (Panel 1). While it is important for a modernized PHS model to be rooted in sustainability and common data principles (FAIR and SMART),^48^^,^^49^ it must be followed with two major priorities for performance and scalability.

Panel 1 **Key recommendations for modernizing public health surveillance for global health security.17,33,38,69**

**Table:** 

Level of action	Stakeholder	Recommendation
Governance	• Government Offices of Pandemic Preparedness and Response Policy • Healthcare and Hospital Services Agencies • Departments or Ministries of Health • Global Health Security Diplomacy Centers • Centers for Disease Control and Prevention • Departments or Ministries of Defense • Departments or Ministries of Finance • Departments or Ministries of Foreign Affairs • International partners (United Nations, World Health Organization [WHO])	• Improve adaptation, implementation, and evaluation of international regulatory frameworks such as the International Health Regulations (IHR 2005), WHO health emergency preparedness, response, and resilience (HEPR) framework, and other global best practices for public health surveillance • Develop and enforce policies that foster the streamlining and interoperability of existing siled systems and processes across collaboration, informatics, and analytics • Create or reprioritize funding laws, regulations, and policies for greater, integrated, flexible, equitable, and sustainable funding • Establish laws and policies to support the systematic scale up of innovative, proven, and cost-effective system and process change interventions
Collaboration	• Governments (federal, states, tribes, localities, and territories) • Health (healthcare and public health) sectors • Non-health sectors • Academic sector • Private sector • Non-Governmental Organizations • Civil societies • Populations	• Support multilevel, multisectoral, and transdisciplinary partnerships for the implementation of public health surveillance interventions across exploration, planning, implementation, and sustainability phases • Promote stakeholder envisioning and capacity building for public health surveillance using communication and coordination best practices (e.g., transparency) • Establish equitable national and transnational collaborations for open data access and exchanges, learning, and adaptation on informatics and analytics to improve public health surveillance
Informatics	• Informaticians • Computer specialists (developer, programmer, licensor) • Information and communication technology specialists • Health (healthcare and public health) practitioners • Non-health practitioners • Policymakers • Populations	• Develop standards-based and interoperable data infrastructure for public health surveillance models with secure, open data access, and exchange capabilities that ethically leverage new technologies • Design, test, and implement public health surveillance models using user-centered and iterative design approaches to improve their contextual fit and usability • Advance public health surveillance models databases with equity datapoints • Embed a monitoring, evaluation, and learning framework into the design and implementation of public health surveillance models
Analytics	• Statistical software specialists (developer, programmer, licensor) • Data scientists (analyst, statistician, managers) • Epidemiologists • Health (healthcare and public health) practitioners • Non-health practitioners • Policymakers • Populations	• Implement robust and equity-focused data analysis and visualizations for public health surveillance models • Develop capabilities for real-time analysis and information sharing for appropriate intelligence to inform public health emergency prevention, preparedness, and response • Establish capabilities for real-time dissemination of public health risks • Facilitate effective risk communication as informed by evidence, including sharing of action steps and success stories

#### Data infrastructure performance and scalability priorities

The first priority is to generate sufficient evidence on its effectiveness and benefits relative to cost and risks after a careful analysis of the technical needs and standards that would be required to enable a full implementation. Further, given potential ethical and technical challenges for randomization and selection of representative controls, an applied pilot study with the hybrid type-1 quasi-experimental design (i.e., having a primary aim of determining the effectiveness of an intervention and a secondary aim of gaining insights into its implementation) should be pursued. The pilot study should seek to determine the baseline evidence on these outcomes for the traditional PHS model that exists in the locality where the study will be conducted (e.g., a county with a distinct PHS system can be identified in a state). Where possible, a five- or 10-year average of the pre-defined indicator(s) for these outcomes per PHS system should be used for all disease-specific outbreaks in the locality of study to improve validity. Thereafter, the effectiveness of the modernized PHS model should be assessed using predefined PHS attributes and indicators from an approved monitoring, evaluation, and learning framework with bold yet realistic targets, including: 1) *completeness*, 2) *validity*, and 3) *timeliness* at the institutional and community levels, as its short-term outcomes.^70^^,^^71^ PHS attributes, including: 4) *sensitivity*, 5) *positive predictive value*, 6) *stability*, and 7) *utility*, should be prioritized as medium-term/long-term effectiveness outcomes for a larger study using the hybrid type-2 design (i.e., having a concurrent evaluation of effectiveness and implementation outcomes).^70^, ^71^, ^72^ Regarding implementation, the study should be designed to evaluate key outcomes, including: 1) *contextual fit*, 2) *feasibility*, and 3) *constraints/enablers*,^73^ as detailed in (Supplementary Panel S1). Additionally, this model can be evaluated against the newly recommended 7-1-7 (i.e., 7 days for outbreak detection, 1 day for notification or reporting, and 7 days for initial emergency response completion) targets for epidemic prevention and followed up with the assessment of its impact on outbreaks-related mortality and morbidity. This knowledge should then be used to inform its refinement for sustainable scale up.^74^^,^^75^ Finally, to ensure real-world impact of this PHS model, these outcomes should be assessed using pragmatic implementation science methodologies, including PRagmatic Explanatory Continuum Indicator Summary (PRECIS-2), Pragmatic Contextual fit and Feasibility (PCoF), and Organization Readiness for Implementing Change (ORIC) tools.^72^^,^^73^^,^^76^

The second priority is to ensure broad dissemination of evidence from the PHS model pilot test and deployment in open-access knowledge domains, including institutional websites, journal publications, and other global public good repositories, such as WHO gdhub for Digital Health Implementations and Insights, to foster widespread scale up and local adaptation (Box 1).^77^

Box 1 **Take home-points.**

• In today's world of increasing human-animal-environment interdependence, health systems vulnerabilities, and economic fragility, traditional public health surveillance (PHS) models are inadequate to address public health risks from climate change, pathogen spillover, and intentional pathogen release to ensure global health security.^11^^,^^12^
• While high-yield evidence-based approaches such as One Health and ethical AI show promises in modernizing these PHS models, progress in their adoption, institutionalization, performance, and scalability is slow and uneven within and between countries, in part, due to weak data infrastructure, misplaced implementation priorities, and limited operational information gaps.^29^, ^31^
• In this Health Policy, we argue that novel multifaceted innovations like a cloud-based, artificial intelligence-enabled One Health Integrated Disease Surveillance and Response health information system (AI-OneHIS) data infrastructure, providing faster data capture, seamless interoperability of fragmented HIS, and precise decision support, and implemented under a strong governance-collaboration-informatics-analytics framework, should be the new standard for benchmarking PHS models.
• This is crucial to reduce the case reporting burden, uncoordinated public health emergency management efforts, and operational cost that continue to overwhelm many health systems around the world.

## Conclusion

To advance GHS, a modernized PHS model with AI-OneHIS data infrastructure providing seamless and secure integration and harmonization of fragmented HIS across human, animal, environment health sectors, while preserving their structures, functionalities, and capabilities for routine operations and data sovereignty, should be the new standard for benchmarking PHS models. Considering, the global collective support for e-PHS, One Health, and the Pandemic Agreement,^3^^,^^9^^,^^78^, ^79^, ^80^, ^81^ we encourage health institutions, countries, and partners to consider and adapt this multifaceted innovation to improve the timeliness, completeness, and accuracy of their PHS models for the prevention, detection and response to PHE. This is particularly important to reduce case reporting burden, uncoordinated PHE management efforts, and operational cost that continue to overwhelm many health systems around the world.

## Contributors

SM, KOO, ATS, AB, MT, and BED led the conceptualization and design of this study. CO, WAG, VKL, DW, and YS contributed to the conceptualization and provided critical suggestions for the design of this study. KOO, AB, and SM performed the formal analysis and wrote the manuscript draft. SA, WAG, NS, SK, RJA, SC, WA, JNS, TW, and MAA performed review of the technical aspects of the manuscript. CO, YS, and SM critically revised the manuscript for important intellectual content. All authors read and approved the submission of the final manuscript.

## Declaration of interests

BED declares grants payment from the U.S. Centers for Disease Control and Prevention (CDC) to his institution to conduct surveillance of infectious diseases, maternal and child health, and immunization safety. Other authors declare no competing interests for this study.

## Appendix A Supplementary data

Supplementary Panel S1
