# Supplementary material for: Modernizing public health surveillance for global health security leveraging AI
Source: Lancet Reg Health Am. 2026 Mar 17;58:101452. doi: 10.1016/j.lana.2026.101452 (PMC13015731; doi:10.1016/j.lana.2026.101452)
Supplement: Supplementary Panel S1 [file mmc1.docx]

**Panel S1.** Minimum attributes and indicators requirement for a monitoring, evaluation, and learning framework for a public health surveillance model.^1-3^

| **Attribute** | **Description** | **Indicator** |
| --- | --- | --- |
| *Completeness*        Internal completeness  External completeness | The match between the number of cases or outbreaks registered (e.g., in record systems) and what is reported without missing values in the public health surveillance (PHS) system or between the number reported and what is detected (e.g., in health systems)   - The match between the number of cases or outbreaks registered and what is reported in the PHS system without missing values (e.g., unknown or blank) - The match between the number of cases or outbreaks reported in the PHS system and what is detected | - Proportion of cases or outbreaks registered in the PHS system with or without missing values that are reported without missing values - Proportion of cases or outbreaks detected in the PHS system that are reported with or without missing values |
| *Validity*    Internal validity  External validity  *Timeliness*    Case/event alert    Case/event reporting      Case/event registration    Case/event detection | - The match between the number of cases or outbreaks registered and what is reported without errors (e.g., misentered, misdiagnosed) in the public health surveillance (PHS) system or between the number reported and what is expected (e.g., from bias adjusted estimations) - The match between the number of cases or outbreaks registered (e.g., in record systems) and what is reported in the PHS system without errors - The match between the number of cases or outbreaks reported in the PHS system and what is expected - The time from when cases or outbreaks are detected through the time they are reported or predefined automated alerts are generated in the public health surveillance system (PHS) - The time from when cases or outbreaks are detected to the time predefined automated alerts are generated in the PHS system - The time from when cases or outbreaks are detected to the time they are reported in the PHS system - The time from when cases or outbreaks are detected in the PHS system to the time they are registered in the record systems - The time from when cases or outbreaks are detected at the health systems or communities to the time of the occurrence of first symptoms/signs of case or first observation of outbreaks emergence | - Proportion of cases or outbreaks registered in the PHS system with or without missing values that are reported without errors - Proportion of cases or outbreaks expected that are reported in the PHS system with or without errors - Average number of days between the time cases or outbreaks are detected to the time predefined automated alerts are generated in the PHS system - Average number of days between the time cases or outbreaks are detected to the time they are reported in the PHS system - Average number of days between the time cases or outbreaks are detected to the time they are registered in the PHS system - Average number of days between the time cases or outbreaks are detected in the PHS system to the time of the occurrence of first symptoms or signs of cases or first observation of outbreaks emergence |
| *Sensitivity*    Case/event detection  Case/event definition | - The ability of the public health surveillance (PHS) system to identify all cases or outbreaks that truly have the disease or event under study - The ability of PHS system to identify all cases or outbreaks detected or estimated that truly have the disease or event under study - The ability of PHS system to identify all cases or outbreaks detected and confirmed or estimated and confirmed (e.g., from predictive modeling) meeting case definitions that truly have the disease or event under study | - Proportion of all cases or outbreaks detected or estimated in healthcare and/or communities that truly have the disease or event - Proportion of all cases or outbreaks detected and confirmed or estimated and confirmed in in healthcare and/or communities that truly have the disease or event |
| *Positive predictive value*  *Stability*  *Utility* | - The ability of the public health surveillance (PHS) system to identify reported cases or outbreaks that truly have the disease or event under study - The ability of the public health surveillance (PHS) system to collect, collate, analyse, and disseminate when it is needed - The use of results from the public health surveillance (PHS) system to inform public health action | - Proportion of reported cases or outbreaks in the PHS system that truly have the disease or event - Proportion of time the PHS system is operating fully - Proportion of public health action informed by results from the PHS system |
|  | | |

**References**

1.. McNabb SJN, Chungong S, Ryan M, et al. Conceptual framework of public health surveillance and action and its application in health sector reform. *BMC Public Health*. 2002;**2**:2.

2. Nsubuga P, White E, Thacker S, et al. Public health surveillance: A tool for targeting and monitoring interventions. In: Jamison D, Breman J, Measham A, et al., editors. Disease control priorities for developing countries. Washington, DC: World Bank. 2006. pp. 997–1015.

3. WHO. Communicable disease surveillance and response systems: guide to monitoring and evaluating. World Health Organization epidemic and pandemic alert and response. 2006. https://iris.who.int/server/api/core/bitstreams/6c51bddd-d792-469a-9fb3-8f4eeb7c0e7c/content (accessed August 17, 2024).
